# Cerebral Vasculitis in Ulcerative Colitis Is Predominantly Venular: Case Report and Review of the Literature

**DOI:** 10.1155/2019/9563874

**Published:** 2019-02-26

**Authors:** Paul T. Parks, Alexander S. Easton

**Affiliations:** ^1^Faculty of Medicine, University of Ottawa, Ottawa, Ontario, Canada; ^2^Department of Pathology, Dalhousie University, Halifax, Nova Scotia, Canada

## Abstract

Extraintestinal complications of ulcerative colitis include isolated case reports of cerebral vasculitis. In this case report, we describe autopsy findings in a 50-year-old female who died as a result of massive multifocal cerebral hemorrhage. Microscopic examination of the left colon showed findings typical for ulcerative colitis. Examination of the brain showed an extensive vasculitis. More affected vessels were noted in grey matter than in white matter. Many showed fibrinoid necrosis, invasion by neutrophils and thrombosis. There was extensive perivascular hemorrhage with associated infarction. Vessel analysis shows most of the vessels to have been venous rather than arterial. There were no perivascular sleeves of demyelination to suggest a primary demyelinating disorder, such as acute hemorrhagic leucoencephalitis. Our analysis shows that veins are the likely target of cerebral vasculitis in ulcerative colitis. This has clinical implications because venous occlusion generally causes massive intracerebral hemorrhage with a high mortality.

## 1. Introduction

Ulcerative colitis (UC) is one of the major forms of inflammatory bowel disease alongside Crohn's disease, which is defined by colonic inflammation. In fact, UC is a multisystem disorder with many extraintestinal manifestations that affect many organs including the joints, skin, eyes, and hepatobiliary system. As such, it captures the attention of a variety of medical specialists including rheumatologists, who manage many forms of pauciarticular, polyarticular, and axial arthropathies, including ankylosing spondylitis and sacroiliitis [1]. Several reviews suggest that neurological complications in UC are underdiagnosed and underreported [2–4]. These complications vary in their incidence in UC depending on study design and which disorder is being studied. Two retrospective observational studies estimated neurological complications in between 1 and 2% of patients with UC [5, 6]. However, peripheral neuropathy was reported in as many as 45.1% of UC patients and headache in 57% of UC patients, in a prospective clinic-based study [7]. Another study that used health records found that peripheral neuropathy occurred in 2.4% of UC patients compared to 1.35% in the general population [8]. Asymptomatic white matter lesions were detected by MRI scan in 45.8% of UC patients compared to 16% of healthy age-matched controls [9]. Most neurological complications occur with active intestinal disease [10], and there is a significant risk posed by more recent biologic therapies including monoclonal antibodies that target tumor necrosis factor, TNF [11]. Anti-TNF therapies have been linked to peripheral neuropathy, multiple sclerosis, and progressive multifocal leukoencephalopathy [3].

There are three major groups of neurological complication in UC. First, there is a risk of stroke, both arterial and venous from cerebral thromboembolism. Second, there is peripheral neuropathy. Third, there may be an increased risk of multiple sclerosis, a major demyelinating disease of the central nervous system [4]. Stroke may be caused by a variety of factors including coagulation disorders, but cerebral vasculitis is a possible contributing factor, particularly when inflamed vessels undergo thrombosis and occlusion. Cerebral vasculitis can occur in patients who develop a lupus-like syndrome after treatment with anti-TNF therapies associated with high serum titres of antibodies against double-stranded DNA [11]. Clinically, cerebral vasculitis in UC may be overdiagnosed [3] because it produces white matter changes on MRI scans that also occur incidentally as mentioned above [9]; however, there have been no systematic studies to define the true incidence of cerebral vasculitis in UC. Few cases have been confirmed by brain biopsy or autopsy. We have identified 6 published case reports on the histopathology of cerebral vasculitis in UC [12–17], and we will compare findings in 4 of these reports [12, 13, 16, 17] with the present case. The focus of this case report is also on the histopathology of cerebral vasculitis in a patient with UC examined at autopsy. What this report adds to those already published is a detailed examination of the type of blood vessel targeted by the disease. We conclude that cerebral vasculitis primarily targets venous rather than arterial vessels, and so can more accurately be described as predominantly a cerebral venulitis. The pathology from previous reports supports this conclusion, which adds to our understanding of the devastating nature of this complication because venous occlusion in the brain usually results in massive internal hemorrhage with a high mortality.

## 2. Case Report

**Figure 1:**
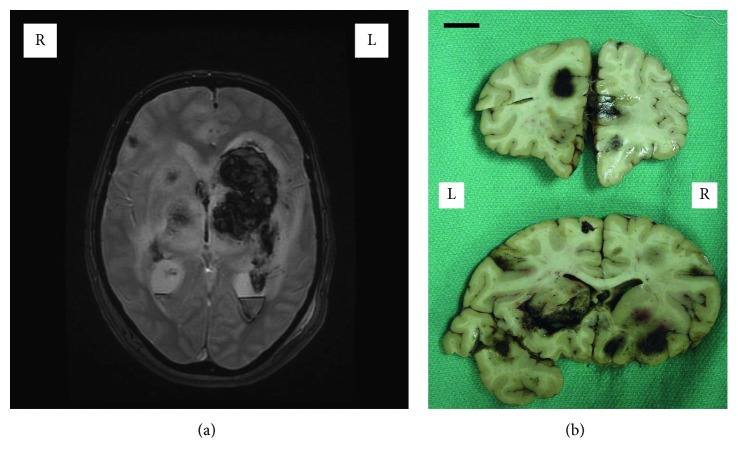
(a) Axial T2-weighted gradient echo magnetic resonance image showing hemorrhage in the left basal ganglia. (b) Gross photograph of coronal sections through the cerebral hemispheres. Scale bar = 1 cm. L = left; R = right.

This case concerns a 50-year-old female. Past medical history was significant for ulcerative colitis, hypertension, hypothyroidism, and a left deep vein thrombosis. She had a history of intermittent night sweats for 3-4 months before presentation. Her ulcerative colitis had flared up about 6 months before presentation to hospital and was treated with prednisone 5 mg daily. This flared again 2 weeks before presentation, so prednisone was restarted, and the day before presentation she underwent colonoscopy. She was on drugs to treat hypertension (ramipril), elevated cholesterol (rosuvastatin), hypothyroidism (levothyroxine), and ulcerative colitis (mesalazine and prednisone 5 mg daily for the recent flare up). She had not been treated with anti-TNF biologics. For her presenting complaint, there was a one-day history of continuous dull headache increasing in severity by evening. The patient then developed slurred speech, unsteady gait, and progressive right-sided weakness. She was seen at a local hospital, where her blood pressure was recorded as 177/95, pulse 90/min, and temperature 35.9°C. Her left pupil was 3 mm in diameter and sluggishly reactive to light, while the right pupil was fixed. Her right side was not moving spontaneously, and a right-sided stroke was diagnosed. While in hospital, there was a sudden reduction in the level of consciousness and a “grand mal” seizure lasting for 2 minutes. While in the local hospital, a CT brain scan showed hemorrhage into the left basal ganglia and left temporal lobe with ventricular extension. Just one CT brain scan was carried out owing to the rapidity of events. The next day, she was transferred to a tertiary care hospital where MRI scan gradient echo mapping highlighted the extent of the hematoma in the left basal ganglia (Figure 1(a)). MRA (magnetic resonance angiography) of the circle of Willis demonstrated normal intracranial vessels without focal stenosis or aneurysmal dilatation. There were no vascular malformations. MRV (magnetic resonance venography) demonstrated normal venous sinuses as well as normal deep veins, ruling out widespread cerebral venous thrombosis as the primary diagnosis. The preferred clinical diagnosis was acute hemorrhagic leucoencephalitis, with viral encephalitis and cerebral vasculitis in the differential. On further examination, there was extensor posturing in the left arm and no movement of the right arm. Pupils were asymmetric (right 5 mm, left 3 mm) and fixed. There was no corneal or doll's eye reflex and no facial asymmetry. Tone and bulk were normal, with no fasciculations. Plantars were unresponsive, with absent knee jerks, but other reflexes were 1+ to 2+ in strength. Blood work during the admission showed an elevated white cell count (17.0 × 10^9^/L, normal range 4–11 × 10^9^/L), with increased lymphocytes and neutrophils), normal platelet count (184 × 10^3^/*µ*l, normal range 150–400 × 10^3^/*µ*l), slightly elevated rheumatoid factor (22.5 IU/ml, normal < 15 IU/ml), and markedly elevated C reactive protein (287 mg/ml, normal < 3 mg/ml). A serological screen for autoantibodies was also carried out. The patient was screened for antinuclear antibodies used to diagnose a number of systemic autoimmune rheumatic diseases such as systemic lupus erythematosus, systemic sclerosis, Sjögren's syndrome, mixed connective tissue disease, and idiopathic inflammatory myopathies. The screen measured antibodies against double-stranded DNA, chromatin, ribosomal P, SS-A/Ro, SS-B/La, centromere B, SM, Sm/RNP, Scl-70, and Jo-1, and was negative. In addition, the patient was screened for antiglomerular basement membrane (GBM) antibodies (which defines anti-GBM disease, a small vessel vasculitis of kidneys and lungs), antimyeloperoxidase, and antiproteinase antibodies (which are also elevated in systemic vasculitides), all of which were found to be negative. The patient was not screened for antibodies against antineutrophil cytoplasmic antibodies (ANCAs) or antiphospholipid antibodies. Blood, urine, and sputum cultures were negative. The patient was started on pulse dose steroids for likely vasculitis, but her clinical status rapidly declined, and she died 2 days after presentation.

**Figure 2:**
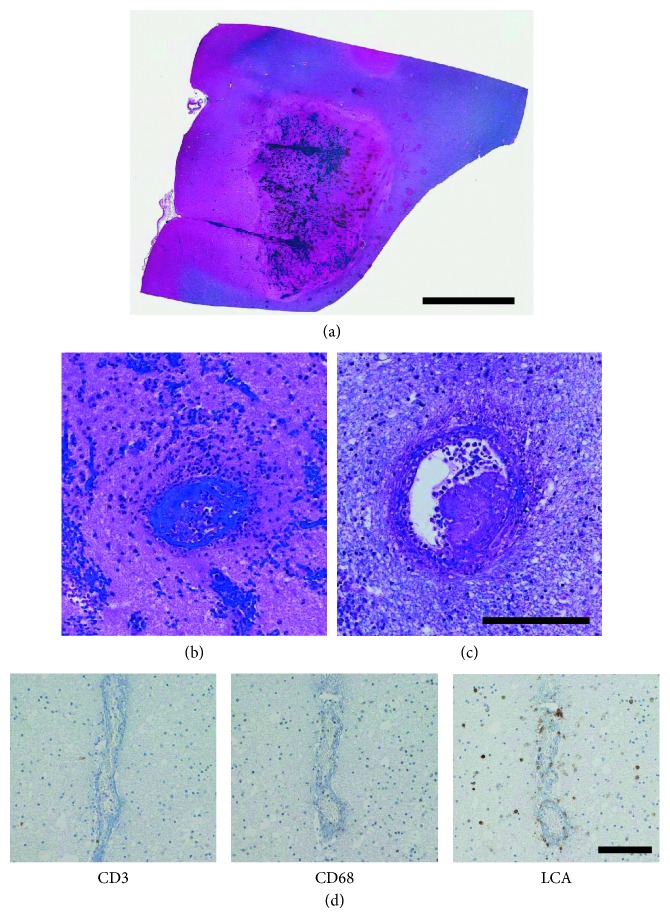
(a) Hemorrhage within the left frontal cortex. (b) Inflamed vessel in the left frontal cortex. (c) Vessel showing intraluminal thrombus and fibrinoid necrosis in white matter adjacent to the left putamen. (d) Immunohistochemistry of vessel in the left putamen, negative for CD3 and CD68 but positive for LCA. Scale bars: (a) 0.5 cm; (b–d) 100 *µ*m.

**Figure 3:**
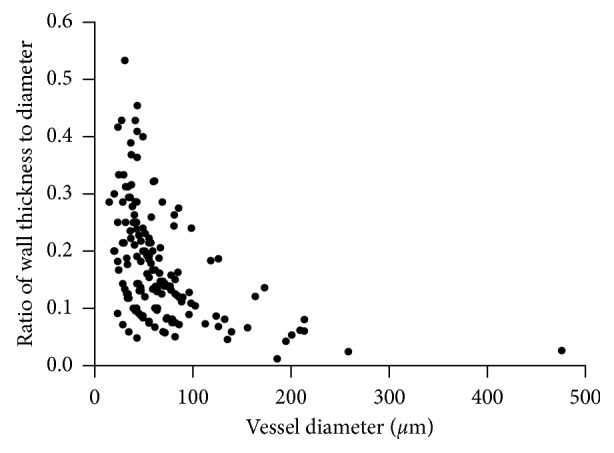
Scatter plot showing ratio of wall thickness to diameter as a function of vessel diameter in 173 pathologically affected blood vessels.

A full autopsy was performed. Continuous mucosal thickening and flattening was noted in the colon, predominantly on the left side, while the esophagus, stomach, duodenum, and ileum had a normal appearance. There was no intestinal hemorrhage, obstruction, or perforation. Microscopic examination of sections from the left colon showed mucosal ulcers with inflammatory exudates. Inflammatory cells including lymphocytes and eosinophils infiltrated the mucosa and submucosa, without vasculitis. There was no inflammation in the muscularis propria or serosa and no evidence of malignancy. These findings were interpreted as “consistent with ulcerative colitis” by the examining anatomic pathologist. When first removed at autopsy, the brain weighed 1400 g before fixation. The fixed brain showed diffuse hemispheric edema and an area of disruption over the left temporal lobe measuring 4.5 × 1.0 cm. There was left-sided uncal herniation, but no tonsillar herniation. Coronal sections through the cerebral hemispheres showed extensive hemorrhage and disruption in the left middle temporal gyrus and inferior portions of the left striatum and pulvinar. There was a discrete hematoma in the left superior frontal gyrus and hemorrhage in the right median frontal cortex and in white matter adjacent to the right orbitofrontal surface (Figure 1(b)). There was extensive right-sided hemorrhage in the brainstem, involving the crus and dorsal pons with extension into the fourth ventricle. Hematoxylin and eosin/luxol fast blue stained sections showed extensive acute hemorrhage with infarction (Figure 2(a)). Changes were noted in both grey and white matter. There was a striking vasculopathy. Vessels showed invasion by leucocytes (Figure 2(b)) as well as fibrinoid necrosis of the wall (Figure 2(c)). Invading leucocytes were rarely positive for the lymphocyte marker CD3 or the macrophage-microglia marker CD68 but more often positive for the general white blood cell marker Leucocyte Common Antigen (LCA/CD45) consistent with neutrophils (Figure 2(d)). Some vessels contained luminal thrombus (Figure 2(c)). There were no perivascular sleeves of demyelination. Detailed measurements were made on 173 involved vessels, of which 88 were in grey matter (cortex, putamen, or brainstem nuclei) and 85 in white matter. The mean diameter (for all vessels) was 66.7 ± 3.9 *µ*m (mean ± standard error of the mean). The ratio of wall thickness to diameter is plotted as a function of vessel diameter in Figure 3. The mean ratio was 0.18 ± 0.01. Most vessels were under 100 *µ*m in diameter which would correspond either to venules or small veins on the venous side or to arterioles or small arteries. However, the ratio of wall thickness to diameter is smaller for venous vessels than arterial ones. It is closer to 0.1 for venous vessels but around 0.5 for arterial vessels [18]. This indicates that most of the involved vessels were venous. The findings point to an acute cerebral vasculitis, predominantly involving invasion of venous vessels by neutrophils.

## 3. Discussion

The main pathologic findings of this study can be summarized as (1) widespread hemorrhagic cerebral infarction, (2) necrotizing cerebral venulitis with intraluminal venous thrombosis, and (3) infiltration of the venous wall by acute inflammatory cells consistent with neutrophils. We have compared these findings to the pathologic descriptions of UC-associated cerebral vasculitis in 4 of 6 published studies and believe that they complement our major finding, which is that UC-associated cerebral vasculitis is predominantly a venulitis rather than an arteritis, affecting cerebral venules or veins to a greater extent than arterioles or arteries. This would predispose patients to venous occlusion and intracerebral hemorrhage with a high mortality.

The ratio of wall thickness to diameter gives a method for describing vessels as likely to be of venous or arterial origin, and Figure 3 demonstrates this point for our case. The vast majority of involved vessels clustered around ratios near to 0.1-0.2, although a few vessels reached ratios of 0.5, and so are likely to be arterial. 4 of the previous 6 histopathological studies of UC-associated vasculitis were reviewed, in order to estimate the ratio for the vessels shown in each publication. 2 vessels have ratios estimated as 0.05 and 0.08 in Figure 2 of Glotzer et al. [12]. In Nelson et al. [13], Figure 2 shows a single vessel with an estimated ratio of 0.13. A single vessel is also shown in Figure 3 of the paper by Unnikrishnan et al. [16] with an estimated ratio of 0.05, and we estimate a ratio of 0.12 for the single vessel shown in Figure 4 of the paper by Raj et al. [17]. The remaining two studies were either inaccessible or had poor quality images. All of these values cluster around the range expected for venous involvement.

Venular forms of cerebral vasculitis have received scant attention in the literature. Primary central nervous system (CNS) vasculitis occurs without evidence of a systemic disorder and mostly targets arterial vessels, existing in granulomatous and nongranulomatous forms. However, a case of pediatric primary CNS vasculitis was reported to be predominantly venular [19]. Venulitis has also been reported in cases of CNS vasculitis associated with Behҫet's syndrome [20] and in some cases of acute multiple sclerosis [21]. Arterial vessels appear to be the target in most other forms of cerebral vasculitis, although histological descriptions vary in quality and accuracy. This case is unlikely to be an unsuspected case of Behҫet's syndrome because the pathology of colitis in Behҫet's syndrome is more akin to Crohn's disease than UC. Typically, cases of Behҫet's syndrome show predominantly right-sided colonic pathology, with large, deep ulcers that penetrate deep into the submucosa. Skip lesions are seen, with sparing of intermediate zones of colonic mucosa. In particular, these cases often show a lymphocytic venulitis [22]. By contrast, cases of UC show predominantly left-sided colonic pathology, with more superficial ulceration, and contiguous mucosal involvement rather than skip lesions. The autopsy findings in this case are therefore consistent with UC rather than Behҫet's syndrome because pathology was predominantly found in the left colon, showed superficial inflammation without vasculitis, and lacked skip lesions.

Although testing was not carried out for antiphospholipid antibodies, a recent paper suggests that positive results are more likely to be seen in cases of Crohn's disease rather than UC [23]. In addition, vascular thrombotic events in cases of Crohn's and UC showed a poor correlation to levels of antiphospholipid antibodies. Even if this patient had shown evidence for elevated antiphospholipid antibodies, the significance of such a finding is therefore obscure.

Two previous reports [12, 14] have equated UC-associated cerebral vasculitis with acute hemorrhagic leucoencephalitis (AHL), but there are clear differences. Acute hemorrhagic leucoencephalitis (AHL) is an acute demyelinating disease of the CNS, characterized by multifocal petechial hemorrhages that predominantly affect white matter, with sparing of cortical grey matter. Affected vessels show sleeves of perivascular demyelination, as well as fibrinoid necrosis and invasion by neutrophils, resulting in perivascular hemorrhages. AHL is considered to be a hyperacute form of acute demyelinating encephalomyelitis (ADEM) in which perivenous demyelination is associated with perivascular cuffs of mononuclear inflammatory cells. In both cases, the suggested immune mechanism is directed against myelin, lending similarities to the demyelinating lesions of multiple sclerosis. Compared to AHL, this case showed no perivascular demyelination, white matter involvement did not predominate, and hemorrhages were extensive instead of mostly petechial. However, like AHL, the vasculitis was venous and acute, with predominant invasion by neutrophils. This suggests that the acute cerebral vasculitis in UC is not part of an immune state that predisposes patients to demyelinating conditions such as multiple sclerosis and ADEM. Venous vessels rather than myelin are the likely target of the immune system, perhaps through antigen-antibody complexes and complement activation, and the resulting damage predisposes to extensive hemorrhage since the necrotic tissue is supplied by vessels under arterial blood pressure.

If vasculitis in UC is venular rather than arterial, this has practical clinical consequences. If patients present with a hemorrhagic stroke, vasculitis/venulitis should be considered in the differential diagnosis, whereas a bland nonhemorrhagic stroke is more likely to be arterial in origin. Where venulitis is considered, it may be possible to initiate anti-inflammatory therapies in addition to more typical treatment modalities for stroke. However, massive intracerebral bleeding of the sort seen in this case is likely to carry a high mortality, regardless of treatment.
